# Sunitinib-associated hyperammonemic encephalopathy successfully managed with higher intensity conventional hemodialysis: A case report

**DOI:** 10.1097/MD.0000000000024313

**Published:** 2021-02-05

**Authors:** Sabrina Haroon, Stephanie Ko, Alvin Wong, Poh-Seng Tan, Evan Lee, Titus Lau

**Affiliations:** a Division of Nephrology; b Division of Advanced Internal Medicine; c Department of Medical Oncology; d Division of Gastroenterology and Hepatology, National University Hospital Singapore, Republic of Singapore.

**Keywords:** hemodialysis, hyperammoniemia encephalopathy, renal cell carcinoma

## Abstract

**Rationale:**

Hyperammonemia encephalopathy is a rare but severe complication that has been reported in association with the use of sunitinib, a tyrosine kinase inhibitor. We report here a unique case of a patient with end stage renal disease that was initiated on sunitinib for metastatic renal cell carcinoma.

**Patient concerns:**

A 65-year-old man with end stage renal disease on maintenance conventional hemodialysis and had concomitant stable Child-Pugh class B liver cirrhosis consequent of hepatitis C infection was started on sunitinib for metastatic renal cell carcinoma. He developed confusion few weeks after starting therapy with no other indication of worsening liver dysfunction otherwise.

**Diagnosis:**

He was later diagnosed with hyperammonemia encephalopathy.

**Interventions:**

His treatment was discontinued and reinitiated at a lower dose after recovery and titrated according to tolerance. As ammonia is a very low molecular weight molecule and is cleared well with diffusive clearance, we intensified his dialysis regimen by increasing intensity for each session and frequency per week.

**Outcomes:**

With this change in dialysis regimen, patient was able to continue treatment with sunitinib.

**Lessons:**

Clinicians prescribing sunitinib should be vigilant to monitor for this complication in patients receiving sunitinib, apart from the more usual presentation of hepatotoxicity. We found that a more intensive hemodialysis regimen consisting of 4× a week conventional high-flux hemodialysis (HD) can permit the continuation of treatment with sunitinib in an end stage renal disease (ESRD) patient with Child-Pugh class B liver cirrhosis.

## 1 Introduction

The incidence of renal cell carcinoma (RCC) in end stage renal disease (ESRD) population is many-fold higher than that of the general population, especially those with acquired cystic kidney disease associated with ESRD.^[1]^ A proportion of these are diagnosed with advanced stage metastatic RCC at the onset. Approximately 80% of RCC is of the clear cell type and with better molecular understanding of the disease biology; various targeted therapies are available today for different disease stages that require systemic therapy beyond surgical excision. Metastatic RCC is difficult to treat and treatment options are usually toxic with poor response rate. The advent of tyrosine kinase inhibitor (TKI) as effective therapy for metastatic RCC resulted from discovery that disruption of the vascular endothelial growth factor (VEGF) signaling pathway can retard tumor growth and progress.^[2]^ Sunitinib has inhibitory effect on many receptor kinases such as VEGF receptors and platelet derived growth factor (PDGF) receptors. It is one of the current recommended evidence-based systemic therapies for metastatic RCC.^[3]^

Sunitinib is approved for use in ESRD patients on hemodialysis (HD) with no initial dose adjustment needed.^[4]^ Sunitinib and its metabolite is also highly protein bound (>90%) and hence, very little is removed during treatment with conventional (high-flux) HD. According to the prescribing information (provided by Food and Drug Administration), the pharmacokinetic of sunitinib is not different in stable patients with Child-Pugh class A and B liver cirrhosis.^[4]^

This purpose of this report to highlight the occurrence of a rare complication arising from the use of sunitinib and how it was managed given the unique situation that the patient was already established on maintenance HD.

The National Healthcare Group Institutional Review Board determined that case report does not meet definition of human-subject research and approval was not required. Written informed consent for patient information to be published was provided by patient.

## 2 Case report

A 65-year-old man who was on maintenance HD for 27 years presented with gross hematuria for which subsequent imaging was highly suggestive of renal malignancy. He underwent left radical nephrectomy as it was a local disease and pathology confirmed RCC. Two years after the nephrectomy, he was unfortunately diagnosed with metastatic RCC. His past history was significant for Child-Pugh class B hepatitis C (HCV) related liver cirrhosis (albumin 28–35 g/L, Bilirubin <34 μmol/L, INR <1.7, presence of small amount of ascites, one past episode of grade 2 encephalopathy). The only episode of hepatic encephalopathy occurred 15 months before the diagnosis of metastatic RCC and was managed successfully with lactulose; he was subsequently started on rifaximin and had remained well since with no recurrence of encephalopathy. After deliberation of the various approaches for treatment, his oncologist considered TKI to be the best treatment option and started him on sunitinib 25 mg daily.

Our patient was brought to the emergency department (ED) with confusion and bilateral asterixis 44 days after initiation of sunitinib (patient was maintained on 25 mg daily throughout this period). After appropriate investigations including computed tomography (CT) of the brain had ruled out other causes, he was managed as for metabolic encephalopathy (grade 2). His serum ammonia level was 170.5 μg/dL at presentation. His symptoms resolved 4 days later (with discontinuation of sunitinib). His serum ammonia was reduced to 48.7 μg/dL. He received the same dialysis schedule during his hospitalization and was discharged to continue with his usual HD regimen (3×/wk and 3.5 h/session).

He was later seen by his oncologist and was started at a lower dose of 12.5 mg sunitinib daily 2 weeks after his discharge, increasing to 25 mg daily 4 weeks after being stable on the lower dose with no complaints. He presented to ED again with similar presentation of confusion and asterixis 28 days later after the increase in dose. His serum ammonia level was significantly elevated again at 176.1 μg/dL pre-dialysis, declining to 68.2 μg/dL post-dialysis. He was dialyzed with a larger surface area high-flux filter (polysulfone 1.6 m^2^) using blood flow rate of 280 mL/min and dialysate flow rate of 500 mL/min for 4 hours. His body weight was 54.5 kg. His confusion improved significantly post dialysis and he recovered the following day. He was maintained on sunitinib 25 mg daily and discharged well with a schedule for more intensive dialysis (higher frequency 4×/wk and with better clearance achieved by increased blood flow, longer duration, and larger filter). He was successfully kept out of hospital with no recurrence of encephalopathy with that dialysis schedule. There were no adverse events and episodes of confusion.

Except for the elevation in serum ammonia level, there were no changes in coagulation profile, bilirubin, or liver enzymes during each of these 2 admissions for encephalopathy. There were also no other precipitating factors for hepatic encephalopathy that was evident in these admissions. He did not experience constipation and was compliant with lactulose and rifaximin. CT brain done during his admission did not reveal structural brain pathology. He does not consume alcohol and did not take traditional Chinese medicine. He was not given any medication that can inhibit CYP3A4. He was also not taking any therapeutic agent that has reported association with hyperammonemia. Patient was jointly managed by a multi-disciplinary team (Liver, Medical Oncology and Nephrology). All management decisions were discussed and made jointly by consensus.

## 3 Discussion

It has been reported that elevations in serum aminotransferase level were common as observed from clinical trials with sunitinib (39% vs 23% in the control arm). Grade 3–4 elevation (>5× the upper limit of normal) occurred in about 2% to 3% of the trial subjects; most reversed with temporary discontinuation and were able to reinitiate at lower doses.^[5]^ Hyperammonemia encephalopathy (HE) is a somewhat distinct entity. Ammonia is a by-product of nitrogen metabolism in human biology. Ammonia is highly toxic to the nervous system and hence, must be prevented via various physiological mechanisms from reaching a level that causes toxicity. The liver is one critical organ for ammonia catabolism by converting it to urea via the urea cycle although other organs such as the muscle and kidneys also have an important role in ammonia homeostasis. Serum ammonia level is profoundly altered in liver failure resulting in hyperammonemia due to the deficient ammonia clearance by the diseased liver and to the development of portal collateral circulation that diverts portal blood with high ammonia content to the systemic blood stream.^[6]^ Ammonia has the ability to cross the blood brain barrier. Hyperammonemia state causes central nervous system (CNS) toxicity by inducing astrocyte swelling resulting from accumulation of intracellular glutamine as a consequent of ammonia detoxification within the astrocytes.^[7]^

HE with the use of sunitinib is a unique condition where there is no other demonstrable cause of hyperammonemia; specifically there is no clinical evidence of new onset or worsening liver disease which is one of the major known etiologies of hyperammonemia. There have been 6 other reported cases of HE with the use of sunitinib in the literature to date.^[8–12]^ This is the first reported in a patient with ESRD on maintenance HD. While the patient was known to have chronic liver cirrhosis associated with hepatitis C infection, his disease was in stable course and he has not had any episode of encephalopathy in the 16 months preceding the use of sunitinib. His metastatic disease did not involve the liver. None of the previously published cases had background of liver cirrhosis although some did have primary or metastatic disease involving the liver. Comparison of the cases (Table 1) revealed different doses of sunitinib exposure as well as number of days before patients manifest symptoms of HE. Most of the cases of HE reported with the use of sunitinib has onset of between 10 and 14 days. Our patient presented with HE 44 and 28 days after initiation of treatment with sunitinib. Although this may have been partly influenced by the dose of sunitinib used, we believe that this delayed manifestation was because the HD had protected the patient from high level of serum ammonia. He did present with HE eventually when his regular HD regimen was no longer able to cope with the generation of serum ammonia. The pathobiology behind this is uncertain. It is probably not related to urea cycle disorder as the patient recovered without the use of nitrogen scavenger or replacement of urea cycle intermediates.

**Table 1:** Summary of characteristics of previously reported cases of hyperammonemia encephalopathy associated with the use of sunitinib.

Case	Tumor	Liver condition and renal function	Sunitinib dose/mg	Days between drug initiation and encephalopathy	Ammonia level, μmol/L	Treatment	Days to recovery
Our patient-1st admission	RCC	CirrhosisALT 20 U/LAST 27 U/LNormal bilirubinESRD on hemodialysis	25	44	122	Dialysis (usual)+ lactulose 3×/d+ Discontinuation of sunitinib	4
Our patient-2nd admission			25	28	126	Dialysis (increased intensity)+ lactulose 3×/d+Continuation of sunitinib	2
Lee et al^[8]^	GIST (small bowel)	Liver metastasesALT 50 U/LAST 79 U/LBilirubin not availableRenal function not available	50	14	150	Lactulose hourly+ Discontinuation of sunitinib	1
Lee et al^[8]^	GIST (caecum)	ALT 53 U/LAST 44 U/LBilirubin not availableRenal function not available	50	10	277	Lactulose hourly+ Discontinuation of sunitinib	1
Shea et al^[9]^	PNET	Liver metastasesALT 43 U/L, AST 53 U/LNormal bilirubinNormal renal function	12.5	14	147	Lactulose (ensure bowel opening 3×/d)+ Discontinuation of sunitinib	1
Pilanci et al^[11]^	RCC	ALT 25 U/LAST 34 U/LNormal bilirubinNo renal insufficiency	50	14	104	Lactulose hourly+ Discontinuation of sunitinib	7
Lipe et al^[12]^	Infiltrating ductal carcinoma of breast with metastatis to liver	Liver metastasisALT 54 U/LAST 100 U/LBilirubin not availableRenal function not available. Urinalysis normal	No details	12	202	Lactulose (frequency not mentioned)+ Discontinuation of sunitinib	12

Hyperammonemia severe enough to cause encephalopathy has to be managed urgently. Lactulose is intensified and supplemented with an oral non-absorbable antibiotic rifaximin. Both these treatments work synergistically to reduce inhibit the growth of ammonia producing bacteria in the gastrointestinal tract.^[13]^ Although reduction in ammonia load is certainly one important strategy, the removal of ammonia via HD is also very effective. Ammonia is not protein bound and much like urea, is a low-molecular-weight molecule (17 g/mol), hence clearance with HD is extremely effective and can reduce serum ammonia level by >50% after a single session (as evident in our case). Given the property of ammonia, the mode of clearance is not critical. Hemodiafiltration (HDF) has little advantage over high-efficiency HD in ammonia removal. Similar to urea clearance, increasing dialyzer (filter) size, blood flow, and dialysate flow will all enhanced the clearance of ammonia.^[14]^ When the decision was made that TKI (sunitinib) was the better option for managing his disease condition, we increased his intensity of HD (both single session efficiency as well as frequency to avoid the 72 hours weekend interval) to permit continuation of sunitinib. Patient was able to maintain treatment with sunitinib for 3 additional months with the intensified dialysis regimen that kept his serum ammonia below 80 μg/dL. Unfortunately, he later developed various infective episodes and decision was made to manage him conservatively with best supportive care only.

This report highlight the fact that a rare but serious drug related complication associated with the use of sunitinib can be managed successfully with intensive outpatient hemodialysis without discontinuation of the medication in a patient who is already on maintenance HD. The limitation is this a single case report in 1 patient. Nevertheless, it provides insight on possible management option for continuation of sunitinib in the case of hyperammonaemia encephalopathy in a hemodialysis patient.

## Author contributions

**Conceptualization:** Sabrina Haroon, Stephanie Ko, Titus Lau.

**Visualization:** Sabrina Haroon, Titus Lau.

**Writing – original draft:** Sabrina Haroon, Stephanie Ko.

**Writing – review & editing:** Sabrina Haroon, Stephanie Ko, Alvin Wong, Tan Poh Seng, Evan Lee, Titus Lau.

## References

[1] Tsuzuki T Iwata H Murase Y Renal tumors in end-stage renal disease: a comprehensive review. Int J Urol 2018;25:780–6. 30066367 10.1111/iju.13759

[2] Levitzki A Klein S My journey from tyrosine phosphorylation inhibitors to targeted immune therapy as strategies to combat cancer. Proc Natl Acad Sci USA 2019;116:11579–86. 31076554 10.1073/pnas.1816012116 PMC6575164

[3] Escudier B Porta C Schmidinger M Renal cell carcinoma: ESMO Clinical Practice Guidelines for diagnosis, treatment and follow-up. Ann Oncol 2019;30:706–20. 30788497 10.1093/annonc/mdz056

[4] Sunitinib Product Information [Internet]; 2015. Available at: http://www.pfizer.com/products/product-detail/sutent. Accessed on December 13, 2019.

[5] Adams VR Leggas M Sunitinib malate for the treatment of metastatic renal cell carcinoma and gastrointestinal stromal tumors. Clin Ther 2007;29:1338–53. 17825686 10.1016/j.clinthera.2007.07.022

[6] Wright G Noiret L Olde Damink SW Interorgan ammonia metabolism in liver failure: the basis of current and future therapies. Liver Int 2011;31:163–75. 20673233 10.1111/j.1478-3231.2010.02302.x

[7] Parekh PJ Balart LA Ammonia and its role in the pathogenesis of hepatic encephalopathy. Clin Liver Dis 2015;19:529–37. 26195206 10.1016/j.cld.2015.05.002

[8] Lee NR Yhim HY Yim CY Sunitinib-induced hyperammonemic encephalopathy in gastrointestinal stromal tumors. Ann Pharmacother 2011;S0735-6747(20)30674-4. 10.1345/aph.1Q038 21954449

[9] Shea YF Chiu WY Mok MY Sunitinib-induced hyperammonaemia in a patient with pancreatic neuroendocrine tumour. J Clin Pharm Ther 2013;38:327–9. 23586819 10.1111/jcpt.12054

[10] Shinde SS Sharma P Davis MP Acute hyperammonemic encephalopathy in a non-cirrhotic patient with hepatocellular carcinoma reversed by arginine therapy. J Pain Symptom Manage 2014;47:e5–7. 10.1016/j.jpainsymman.2014.01.002 24731681

[11] Pilanc KN Elbüken F Ordu Ç A rare case of sunitinib-induced hyperammonemic encephalopathy and hypothyroidism in metastatic renal cell carcinoma. Am J Ther 2016;23:e583–7. 24901901 10.1097/MJT.0b013e3182a32e0e

[12] Lipe DN Hoxha B Sahai SK Sunitinib-associated hyperammonemic encephalopathy. Am J Emerg Med 2020;10:S0735–6757. 10.1016/j.ajem.2020.07.079 32811710

[13] Bass NM Mullen KD Sanyal A Rifaximin treatment in hepatic encephalopathy. N Engl J Med 2010;362:1071–81. 20335583 10.1056/NEJMoa0907893

[14] Gupta S Fenves AZ Hootkins R The role of RRT in hyperammonemic patients. Clin J Am Soc Nephrol 2016;11:1872–8. 27197910 10.2215/CJN.01320216 PMC5053785

